# Cross verification of independent dose recalculation, log files based, and phantom measurement‐based pretreatment quality assurance for volumetric modulated arc therapy

**DOI:** 10.1002/acm2.13036

**Published:** 2020-10-01

**Authors:** Ce Han, Jinling Yi, Kecheng Zhu, Yongqiang Zhou, Yao Ai, Xiaomin Zheng, Congying Xie, Xiance Jin

**Affiliations:** ^1^ Department of Radiation and Medical Oncology The First Affiliated Hospital of Wenzhou Medical University Wenzhou China; ^2^ Department of Radiation and Medical Oncology The Second Affiliated Hospital of Wenzhou Medical University Wenzhou China

**Keywords:** cross verification, independent dose check, log files based QA, phantom measurement‐based QA, volumetric modulated arc therapy

## Abstract

Independent treatment planning system (TPS) check with Mobius3D software, log files based quality assurance (QA) with MobiusFX, and phantom measurement‐based QA with ArcCHECK were performed and cross verified for head‐and‐neck (17 patients), chest (16 patients), and abdominal (19 patients) cancer patients who underwent volumetric modulated arc therapy (VMAT). Dosimetric differences and percentage gamma passing rates (%GPs) were evaluated and compared for this cross verification. For the dosimetric differences in planning target volume (PTV) coverage, there was no significant difference among TPS vs. Mobius3D, TPS vs. MobiusFX, and TPS vs. ArcCHECK. For the dosimetric differences in organs at risks (OARs), the number of metrics with an average dosimetric differences higher than ±3% for TPS vs Mobius3D, TPS vs MobiusFX, and TPS vs ArcCHECK were 1, 1, 7; 2, 1, 4; 1, 1, 5 for the patients with head‐and‐neck, abdomen, and chest cancer, respectively. The %GPs of global gamma indices for Mobius3D and MobiousFX were above 97%, while it ranged from 92% to 96% for ArcCHECK. The %GPs of individual volume‐based gamma indices were around 98% for Mobius3D and MobiousFX, except for γPTV for chest and abdominal cancer (88.9% to 92%); while it ranged from 86% to 99% for ArcCHECK. In conclusion, some differences in dosimetric metrics and gamma passing rates were observed with ArcCHECK measurement‐based QA in comparison with independent dosecheck and log files based QA. Care must be taken when considering replacing phantom measurement‐based IMRT/VMAT QA.

## 1 INTRODUCTION

The inherent complexity and inverse optimization features of intensity‐modulated radiotherapy (IMRT) require a standard quality assurance (QA) procedure to ensure an accurate delivery of expected dose distribution in patients.^1^ A report from imaging and radiation oncology core (IROC) demonstrated that there was approximately 10% to 23% delivery failure during a basic head‐and‐neck IMRT QA using a 7%/4 mm to 5%/4 mm acceptability criterion, which clearly indicated the challenge and necessity of pretreatment QA for IMRT.^2^ As a novel IMRT delivery technique, volumetric modulated arc therapy (VMAT) has more degrees of freedom by simultaneously moving multileaf collimators (MLCs) and gantry, as well changing the dose rate, which also renders it sensitive to calculation and delivery errors and requires more intensive QA procedures.^3^

Traditionally, the pretreatment IMRT QA was carried out by irradiating a phantom‐detector combination to measure the consistency between delivered and calculated dose distribution.^1^ However, studies pointed out that phantom measurement QA may not be able to detect some types of failures in the IMRT process, such as dose calculation errors, plan transfer errors, etc.^4^ Additionally, the use of water equivalent phantoms for dose recalculation and delivery oversimplifies the QA processes, because water equivalent phantoms do not represent patients' real geometry and tissue heterogeneities.^5^ Another shortcoming of measurement‐based QA is its labor‐intensive and time‐consuming characteristics, and requires access to the treatment machine.

A growing interest in using machine log files and independent treatment planning system (TPS) dose recalculation for IMRT QA has been proposed.^6^, ^7^, ^8^, ^9^ It has been reported that linear accelerator (Linac) log files based QA is able to provide insight into machine parameters that was not possible with phantom‐based QA and to improve the efficiency of patient‐specific QA.^10^, ^11^ Additionally, log files based QA could assess the actual delivered dose by reconstructing the dose on patients' original computer tomography (CT) image sets.^12^ However, the accuracy of log files based QA has also been questioned and concerned.^13^ It has been reported that in some cases the recorded MLC position in the log files did not agree with the observed positions.^14^ Currently, it is still no consensus on whether the Linac log files and independent TPS dose checks are effective enough to be alternative to phantom measurement‐based QA. The purpose of this study was to investigate the dosimetric agreement among independent TPS dose recalculation, log file‐based, and phantom measurement‐based QA in patients who underwent VMAT at different tumor sites.

## 2 MATERIALS AND METHODS

### 2.A Patients and treatment planning

Patients who underwent VMAT treatments from January 2019 to June 2019 were randomly selected and enrolled in this study. One‐arc or two‐arc VMAT plans were optimized with the SmartArc algorithm in the Pinnacle TPS (Philips Healthcare,Fitchburg, WI) for a 6‐MV X‐ray beam. One‐arc plans were optimized with a gantry angle from 181° to 180°. For two‐arc plans, the first arc rotated clockwise from 181° to 180°, and the second arc rotated counterclockwise from 180° to 181°. The collimator was set 15° for all plans. A maximum leaf motion constraint of 0.46 cm/deg and a final arc space of 4 degree were set for both one‐arc and two‐arc VMAT plans with a dose grid of 3 mm × 3 mm × 3 mm during the VMAT optimization. Detailed target volume delineations and optimization parameters were reported in previous studies.^15^, ^16^, ^17^ All plans were delivered on an Elekta Synergy linac(Elekta Ltd,Crawley, UK) with a MOSAIQ record and verify system (version1.60Q3, IMPAC Medical Systems, Inc., Sunnyvale, CA).

### 2.B Independent dose check

Mobius3D software (Mobius Medical Systems, Houston, TX) was applied in this study to verify the VMAT plans generated by Pinnacle TPS independently using a collapsed cone convolution/superposition algorithm.^18^, ^19^ Before the clinical application, the Mobius3D software was commissioned with measured percent depth doses (PDD) and profiles for field sizes 4 × 4–40 × 40 cm^2^, adjusted the parameters of our Linac beam models carefully adjusted to scale the model correctly. The accuracy of the model was verified using Mobius Verification Phantom™ (MVP**).** After the generation of VMAT plans in Pinnacle, DICOM data of the plans (CT images, RTPlan, RTStructure, and RTDose) were exported into the Mobius3D software for recalculation. The PTV prescription doses were 50 Gy/25fx for head‐and‐neck, 60 Gy/30fx for chest, 45 Gy/25fx for abdomen, respectively.

### 2.C Log files based QA

MobiusFX (Mobius Medical Systems LP, Houston, TX) is able to use log files to access the delivery accuracy of IMRT/VMAT plans. After the treatment delivery, machine log files recorded the actual linac delivery information which contains the MLC positions, gantry, dose rate, MU, control points, etc. In this study, the machine log files were generated during the treatment delivery for real patients rather than in QA mode. Then, the log files were uploaded to MobiusFX for dose reconstruction. After the automatic background calculation, we could not only obtain the 3D dose distribution under the actual treatment state of the accelerator, but also 3D plan percentage gamma passing rate (%GPs) and individual volume‐based 3D gamma passing rates.^20^

### 2.D Phantom measurement‐based QA

Phantom measurement‐based QA was carried out by a 3D diode array ArcCHECK phantom (Model 1220; Sun Nuclear Corporation, USA). During phantom dosimetric verification, an ArcCHECK movie (ACML) file was generated containing calculated gantry angles as a function of time. The reconstructed dose distributions were generated by a 3DVH program (Sun Nuclear Corporation, USA) using a planned dose perturbation (PDP) algorithm with the ACML files, RTPlan, RTstructure, and RTDose exported from TPS. Both global gamma index and individual volume‐based gamma index were applied for QA analysis. Detailed phantom‐based pretreatment VMAT QA with ArcCHECK and 3DVH had been reported in previous studies.^20^, ^21^

### 2.E Evaluation parameters and statistical analysis

The dosimetric evaluation parameters for target coverage and OARs sparing comparison were extracted from dose–volume histograms (DVHs). Target parameters, such as Dmax, Dmean, V95, V100 (percentage of the volume irradiated by 95% and 100% of the prescription dose), D2, and D98 (dose irradiated to 2% and 98% of the volume) of the PTV were extracted and compared. For OARs sparing comparison, different Dx and Vx parameters were extracted and compared for different cancer sites. Variables were summarized as mean values with standard deviations. All the statistical analyses were performed with SPSS 22.0(spss Inc., Chicago, IL, USA). A *P* < 0.05 is considered statistically significant and all reported *P* values are two‐sided.

## 3 RESULTS

**Fig. 1:**
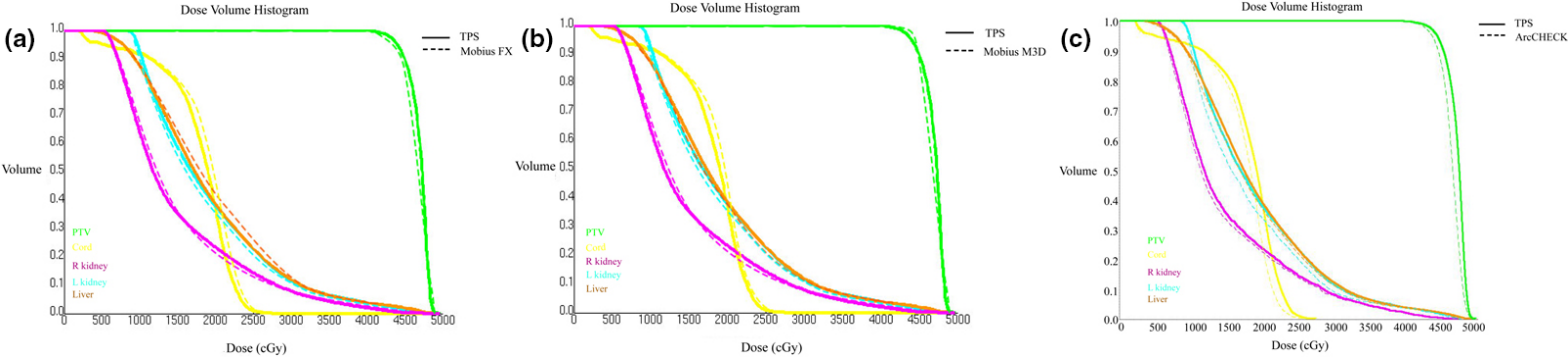
A typical dose–volume histogram comparison for abdominal cancer, (a) treatment planning system (TPS) vs Mobius FX; (b) TPS vs Mobius M3D; (c) TPS vs ArcCHEKC

A total of 52 patients with cancers in head‐and‐neck (17 patients), chest (16 patients), and abdomen (19 patients) underwent VMAT treatment were enrolled in this study. The dosimetric differences resulted from TPS vs independent dose check with Mobius3D, TPS vs reconstructed dosimetric distribution with MobiusFX QA, and TPS vs. ArcCHECK‐based measurement QA, were analyzed and reported using ANOVA. Figure 1 shows one typical DVH comparison of one abdominal cancer patients. Detailed dosimetric differences for head‐and‐neck, chest, and abdominal cancer patients were shown in Tables 1, 2, and 3, respectively.

**Table 1:** Percentage dosimetric differences between treatment planning system (TPS) vs M3D, TPS vs MFX, and TPS vs ArcCHECK for head‐and‐neck cancer patients.

Metrics	Dosimetric differences (%)			*P*		
	M3D	MFX	ArcCHECK	M3D vs MFX	M3D vs ArcCHECK	MFX vs ArcCHECK
Planning target volume						
Dmax	0.41 ± 0.97	0.54 ± 1.33	1.17 ± 1.87	0.82	0.19	0.27
Dmean	−0.02 ± 0.93	−0.05 ± 1.01	−1.33 ± 1.85	0.95	0.02	0.02
V95	−0.28 ± 1.53	−0.39 ± 1.57	−1.94 ± 1.63	0.86	0.01	0.02
V100	−2.56 ± 2.67	−2.06 ± 2.19	−2.77 ± 2.64	0.62	0.83	0.48
D98	−0.14 ± 2.84	−0.29 ± 2.73	−2.49 ± 1.76	0.88	0.02	0.03
D2	0.33 ± 0.99	−1.44 ± 6.24	0.15 ± 2.02	0.25	0.91	0.29
Left parotid						
D50	−0.50 ± 2.93	−0.69 ± 2.96	−5.68 ± 3.51	0.88	<0.001	<0.001
Dmean	−0.93 ± 1.44	−1.03 ± 1.73	−3.08 ± 2.91	0.90	0.01	0.02
Right parotid						
D50	−0.50 ± 2.93	−0.69 ± 2.96	−5.68 ± 3.51	0.88	<0.001	<0.001
Dmean	−0.93 ± 1.44	−1.03 ± 1.73	−3.08 ± 2.91	0.90	0.01	0.02
Brainstem						
Dmax	−2.14 ± 1.45	−0.11 ± 6.59	−0.59 ± 3.42	0.24	0.37	0.78
D1	−2.08 ± 1.87	−1.81 ± 1.92	−3.24 ± 2.96	0.77	0.21	0.14
Cord						
Dmax	−0.64 ± 2.21	−0.20 ± 2.53	−4.07 ± 3.11	0.67	0.002	0.001
D1	1.86 ± 2.97	1.42 ± 2.29	−3.21 ± 2.86	0.68	<0.001	<0.001
Lens						
Dmax	11.45 ± 10.65	9.87 ± 7.95	0.17 ± 11.78	0.70	0.01	0.02

Notes

TPS: treatment planning system; M3D: Mobius3D software; MFX: MobiusFX software

**Table 2:** Percentage dosimetric differences between TPS vs M3D, TPS vs MFX, and TPS vs ArcCHECK for chest cancer patients.

Metrics	Dosimetric differences (%)			p		
	M3D	MFX	ArcCHECK	M3D vs. MFX	M3D vs. ArcCHECK	MFX vs. ArcCHECK
Planning target volume						
Dmax	1.60 ± 1.31	1.78 ± 1.21	0.82 ± 1.91	0.75	0.18	0.10
Dmean	−0.23 ± 1.29	−0.21 ± 1.28	−1.18 ± 1.58	0.96	0.08	0.07
V95	−2.49 ± 2.58	−2.57 ± 2.61	−2.27 ± 3.25	0.95	0.83	0.78
V100	−5.94 ± 5.25	−4.00 ± 2.65	−4.92 ± 3.86	0.21	0.51	0.56
D98	−2.87 ± 3.30	−2.73 ± 3.17	−2.00 ± 2.81	0.91	0.47	0.54
D2	1.23 ± 1.23	1.27 ± 1.24	0.03 ± 1.50	0.95	0.02	0.02
Lung						
V5	0.06 ± 2.77	0.24 ± 2.75	−1.03 ± 2.15	0.86	0.27	0.20
V10	−0.22 ± 1.51	−0.06 ± 1.44	−2.79 ± 2.14	0.81	<0.001	<0.001
V13	−0.61 ± 1.46	−0.44 ± 1.43	−3.38 ± 1.90	0.78	<0.001	<0.001
V20	−2.29 ± 1.41	−2.24 ± 1.58	−4.09 ± 1.67	0.92	0.004	0.003
V30	−4.28 ± 2.29	−4.51 ± 2.59	−4.41 ± 2.07	0.79	0.88	0.91
Dmean	−1.35 ± 1.16	−1.16 ± 1.08	−3.04 ± 1.74	0.73	0.002	0.001
Heart						
V30	−1.64 ± 1.61	−1.62 ± 1.56	−1.96 ± 1.48	0.97	0.59	0.57
V40	−2.95 ± 3.02	−2.97 ± 2.97	−1.94 ± 2.67	0.99	0.36	0.35
Dmean	0.29 ± 1.24	0.35 ± 1.14	−2.85 ± 2.03	0.92	<0.001	<0.001
Cord						
Dmax	−0.54 ± 1.96	−0.01 ± 1.78	−2.41 ± 2.40	0.50	0.004	0.003
D1	0.85 ± 2.11	1.29 ± 1.94	−3.13 ± 2.96	0.60	<0.001	<0.001

Notes

M3D, Mobius3D software; MFX, MobiusFX software; TPS, treatment planning system.

**Table 3:** Percentage dosimetric differences between TPS vsM3D, TPS vs MFX, and TPS vs ArcCHECK for abdomen cancer patients.

Metrics	Dosimetric differences (%)			*P*		
	M3D	MFX	ArcCHECK	M3D vs MFX	M3D vs ArcCHECK	MFX vs ArcCHECK
Planning target volume						
Dmax	2.47 ± 0.96	2.79 ± 1.07	2.38 ± 1.23	0.47	0.83	0.35
Dmean	1.44 ± 0.94	1.66 ± 1.48	−0.31 ± 1.43	0.67	0.002	<0.001
V95	−0.98 ± 1.16	−1.09 ± 1.15	−1.13 ± 1.75	0.84	0.79	0.95
V100	−0.47 ± 2.45	−0.74 ± 2.69	−3.46 ± 2.33	0.79	0.01	0.02
D98	‐1.58 ± 1.52	−1.80 ± 1.93	−0.88 ± 2.32	0.78	0.36	0.24
D2	2.50 ± 0.79	2.92 ± 1.57	1.29 ± 1.38	0.42	0.02	0.003
Left kidney						
V15	3.29 ± 2.47	2.96 ± 3.56	−3.90 ± 5.24	0.83	<0.001	<0.001
V20	3.15 ± 5.09	1.82 ± 6.06	−2.33 ± 7.61	0.60	0.03	0.10
Dmean	0.85 ± 1.17	0.95 ± 1.19	−2.62 ± 1.78	0.86	<0.001	<0.001
Right kidney						
V15	1.35 ± 4.38	0.87 ± 5.00	−4.36 ± 2.44	0.77	0.001	0.002
V20	−0.46 ± 6.25	−0.25 ± 5.48	−3.74 ± 3.55	0.92	0.12	0.10
Dmean	1.55 ± 1.81	1.58 ± 1.74	−2.38 ± 3.21	0.98	<0.001	<0.001
Liver						
V30	2.38 ± 3.34	2.41 ± 3.47	−1.93** **±** **3.92	0.98	0.004	0.004
Dmean	0.88 ± 1.01	0.97 ± 1.15	−0.45 ± 2.35	0.88	0.04	0.03
Cord						
Dmax	2.27 ± 2.11	2.77 ± 2.19	−0.17 ± 4.03	0.66	0.02	0.01
D1	3.04 ± 2.10	3.73 ± 2.90	−1.01 ± 4.75	0.61	0.001	0.001

Notes

M3D, Mobius3D software; MFX, MobiusFX software; TPS, treatment planning system.

For the dosimetric differences in PTV coverage, there was no significant difference among TPS vs Mobius3D, TPS vs MobiusFX, and TPS vs ArcCHECK for patients with head‐and‐neck cancer. The difference in V100 of PTV for patients with abdominal cancer was about −3.46% ±2.33% for TPS vs ArcCHECK, no significant difference in other PTV metrics was observed for abdominal cancer for TPS vs Mobius3D, and TPS vs MobiusFX. For patients with chest cancer, the dosimetric differences in V100 of PTV were −5.94% ± 5.25%, −4.00% ± 2.65%, and −4.92% ± 3.86% for TPS vs Mobius3D, TPS vs MobiusFX, and TPS vs ArcCHECK, respectively.

For the dosimetric differences in OARs, the number of metrics with an average dosimetric differences higher than ±3% for TPS vs. Mobius3D, TPS vs MobiusFX, and TPS vs ArcCHECK were 1, 1, 7; 2, 1, 4; 1, 1, 5 for the patients with head‐and‐neck, abdomen, and chest cancer, respectively. Only for head‐and‐neck cancer patients, there were 1, 1, and 3 metrics with an average dosimetric difference higher than ±5% for TPS vs. Mobius3D, TPS vs MobiusFX, and TPS vs ArcCHECK, respectively. The average %GPs of global gamma indices were 98.0% ± 0.7%, 98.0% ± 0.7%, 92.5% ± 2.7%; 98.9% ± 1.1%, 98.7% ± 1.2%, 94.4% ± 3.6%; and 98.8% ± 1.1%, 98.2% ± 1.3%, 96.3% ± 3.4% for Mobius3D, MobiusFX, and ArcCHECK in patients with head‐and‐neck, chest, and abdominal cancer, respectively. The average %GPs of individual volume‐based gamma indices arranged from 97.3% ± 1.9% to 100% ± 0.1%, 97.3% ± 1.7% to 100% ± 0.1%, 86.2% ± 5.3% to 99.1% ± 1.7%; 92.0% ± 4.5% to 99.5% ± 1.5%, 91.6% ± 4.1% to 99.3% ± 1.8%, 88.4% ± 9.0% to 96.1% ± 3.2%; and 90.4% ± 9.8% to 99.8% ± 0.2%, 88.9% ± 9.8% to 99.9% ± 0.3%, 93.4% ± 5.1% to 97.5% ± 3.2% for Mobius3D, MobiusFX, and ArcCHECK in patients with head‐and‐neck, chest, and abdominal cancer, respectively. Detailed %GPs results are shown in Table 4.

**Table 4:** Percentage gamma pass rates of individual volume gamma indices and global gamma indices for three sites patients at 3%/3 mm criterion.

Gamma index	M3D (%)	MFX (%)	ArcCHECK (%)
Head‐and‐neck			
γPTV	97.3 ± 1.9	97.3 ± 1.7	90.6 ± 5.2
γLeft parotid	99.6 ± 0.9	99.5 ± 1.1	93.6 ± 4.0
γRight parotid	99.7 ± 0.5	99.6 ± 0.4	92.7 ± 3.8
γBrainstem	100.0 ± 0.1	100.0 ± 0.1	93.0 ± 3.9
γCord	98.7 ± 2.0	99.2 ± 0.9	86.2 ± 5.3
γLens	99.0 ± 2.6	99.5 ± 1.1	99.1 ± 1.7
Global gamma	98.0 ± 0.7	98.0 ± 0.7	92.5 ± 2.7
Chest			
γPTV	92.0 ± 4.5	91.6 ± 4.1	90.0 ± 7.7
γLung	99.2 ± 1.3	99.1 ± 1.2	95.0 ± 4.0
γHeart	99.4 ± 1.4	99.5 ± 0.8	96.1 ± 3.2
γCord	99.5 ± 1.5	99.3 ± 1.8	88.4 ± 9.0
Global gamma	98.9 ± 1.1	98.7 ± 1.2	94.4 ± 3.6
Abdomen			
γPTV	90.4 ± 9.8	88.9 ± 9.8	93.4 ± 5.1
γLeft kidney	99.8 ± 0.4	99.9 ± 0.3	97.0 ± 2.7
γRight kidney	99.8 ± 0.4	99.8 ± 0.4	97.1 ± 3.1
γLiver	99.8 ± 0.2	99.7 ± 0.5	97.5 ± 3.2
γCord	99.8 ± 0.5	99.4 ± 1.0	93.7 ± 6.3
Global gamma	98.8 ± 1.1	98.2 ± 1.3	96.3 ± 3.4

Notes

M3D, Mobius3D software; MFX: MobiusFX software.

## 4 DISCUSSION

In this study, multiple pretreatment QA methods: independent TPS dose check, log files based QA and ArcCHECK‐based measurement QA, were performed and compared for 52 patients who underwent VMAT. The dosimetric differences resulted from independent TPS dose check, log files based QA, and ArcCHECK measurement‐based QA were similar, although measurement‐based QA showed several more metrics with larger errors. Gamma indices verifications showed consistent results.

The interests of using independent dose recalculation as an alternative pretreatment IMRT QA method are still on growing. Dietmar et al proposed a semi‐analytic fluence‐based dose calculation to verify the monitor unit (MU) of IMRT plans and achieved an average deviation of 0.5% ± 1.1% and 1.1% ± 2.9% for high dose region and individual beams, respectively.^8^ Acceptable agreement between TPS‐ and Monte Carlo‐based calculation had also been reported.^22^ In this study, the dosimetric differences achieved by independent dose check with Mobius3D were relatively small. Except for V100 of PTV for chest cancer and Dmax of Lens for the head‐and‐neck cancer patients, no other metrics showed a dosimetric difference higher than ±5%. Similar results had been reported in a previous study, in which COMPASS system (version 1.2, IBA Dosimetry, Schwarzenbruck, Germany) was applied for independent TPS check and reasonable dosimetric accuracy was achieved except for D1 of lens for nasopharyngeal cancer patients.^20^ Tyagi et al also observed dosimetric differences up to −21.4% between TPS and independent dose check with 3DVH for VMAT plans.^23^ The relative high errors in Dmax of lens may be due to the relative small volume of lens (0.03 cc) which renders it very sensitive to small spatial deviation between two calculation systems. Similarly, V100 is in a region of great dose gradient which also renders it sensitive to small spatial deviations.

The dosimetric differences resulted from independent dose check and log files based QA were quite consistent in this study. There were no significant differences between TPS vs Mobius3D and TPS vs MobiusFX for these three sites of cancer patients. The same Mobius3D model was used in MobiousFX to reconstruct the dose on patients CT with delivered log files.^24^ However, although most of the relative dosimetric differences were small, significant differences were observed in a few numbers of metrics between Mobius3D vs ArcCHECK and MobiusFX vs ArcCHECK. This was a bit different from a previous study, in which strong coincidence between doses estimated by log files based system and the ionization chamber/ArcCHECK‐3DVH software was observed.^25^ Similarly, Song et al demonstrated that although the dose reconstructed from MobiusFX and 3DVH were not identical, they were generally similar to each other in their verification for 10 prostate, 10 head‐and‐neck, and 10 chest cancer patients who underwent VMAT.^26^

Gamma index evaluation results further demonstrated that independent dose check with Mobius3D and log files based QA with MobiousFX showed a higher consistence with TPS than ArcCHECK‐based measurement did, as shown in Table 4. The %GPs of global gamma indices for Mobius3D and MobiousFX were above 97%, while it ranged from 92% to 96% for ArcCHECK. The %GPs of individual volume‐based gamma indices were calculated with the acceptance criteria: 3%/3 mm, 10% lower dose threshold (such as γPTV, γbrainstem etc.).They were around 98% for Mobius3D and MobiousFX, except for γPTV for chest and abdominal cancer patients (88.9% to 92%); while the %GPs of individual volume‐based gamma indices were variable for ArcCHECK (from 86% to 99%). These deviations could be partly due to the limitation of 3DVH software, which uses preloaded kernel libraries for various Linacs with intrinsic discrepancies, as pointed out by Tyagi et al.^23^ It could also partly due to the output variations during treatment delivery which was not considered in log files.^26^

Although independent TPS check and log files based QA achieved higher accuracy than phantom measurement‐based QA for VMAT patients. DVH metrics with relative high dosimetric errors were still observed for both independent TPS check and log files based QA. Care much be taken when considering replacing the phantom measurement‐based QA for VMAT/IMRT. The accuracy of independent check and log files need further verification. The daily performance of Linac, especially the MLC positioning accuracy should be assured when abandoning measurement‐based QA for VMAT/IMRT.

## 5 CONCLUSIONS

Cross verification of independent dose check, log files based QA and phantom measurement‐based QA showed reasonable accuracy for VMAT in the head‐and‐neck, chest and abdominal cancer patients. Some differences in dosimetric metrics and gamma passing rates were observed with ArcCHECK measurement‐based QA in comparison with independent dose check and log files based QA. Care must be taken when considering replacing the phantom measurement‐based QA for IMRT/VMAT.

## CONFLICT OF INTEREST

The authors have declared that no competing interest exists.
